# Tumour associated tissue eosinophilia as a predictor of locoregional recurrence in oral squamous cell carcinoma

**DOI:** 10.4317/jced.51610

**Published:** 2015-02-01

**Authors:** Nagaraju Rakesh, Yashoda Devi, Kuhu Majumdar, Sujatha S. Reddy, Kunal Agarwal

**Affiliations:** 1 MDS, Ph.D, Reader, Dept. of Oral medicine, Diagnosis and Radiology, M.S. Ramaiah Dental College & Hospital, Bangalore; 2 BK MDS, Professor, Dept. of Oral medicine, Diagnosis and Radiology, M.S. Ramaiah Dental College & Hospital, Bangalore; 3 BDS, MDS, Post graduate student, Dept of Oral medicine, Diagnosis and Radiology, M.S.Ramaiah Dental College & Hospital, Bangalore; 4 MDS, Ph.D, Professor, Dept of Oral medicine, Diagnosis and Radiology, M.S.Ramaiah Dental College & Hospital, Bangalore; 5 MDS, Senior resident, SCB Dental College and Hospital, Odisha

## Abstract

Objectives: The increasing global burden of oral cancer has driven much of the focus of research to the determination of reliable prognostic markers which may have significant effects on survival and the control of post-treatment morbidity. This study was undertaken to evaluate tumour associated tissue eosinophilia (TATE) quantitatively in oral cancer specimens and observe for its possible association with tumour stage, patterns of locoregional recurrence and overall prognosis. Study Design: 14 patients undergoing surgical resection for primary oral squamous cell carcinoma (OSCC) were subjected to grey scale ultrasonography (USG) to assess tumour dimensions. The findings were compared with the cTNM stage initially documented. TATE was evaluated along the invasive tumour front (ITF) using H & E stained sections of histopathological specimens for 10 continuous high power fields (HPF) and graded as mild, moderate or intense. Patients were followed up over 5 years and observed for patterns of recurrence. Results: Loco regional recurrence was significantly associated with intense degree of TATE. (p<0.001) cTNM stage as well as USG stage did not correlate with the degree of TATE with p=0.419 and 0.772 respectively. None of the patients with mild/ moderate dysplasia developed locoregional recurrence within the period of follow up. Conclusions: Analysis of TATE in OSCC patients may provide an early indication of future locoregional recurrence. Identification of an appropriate biopsy site representing the ITF where TATE analysis can be performed may be a simple, inexpensive method of obtaining valuable prognostic information at the time of diagnosis.

** Key words:**Tumour associated tissue eosinophilia, oral cancer, prognosis.

## Introduction

With an annual estimated incidence of around 275,000 and two-thirds of the cases occurring in developing countries alone, oral cancer is a matter of serious concern in these parts of the world. Oral squamous cell carcinoma (OSCC) accounts for more than 90% of all oral cavity cancers (1). Poorer prognosis and survival rates for oral cancer in developing countries can be largely attributed to presentation at a late stage of the disease by patients and failure to follow up cases following treatment (2,3). Socioecnomic concerns, lack of awareness and difficulty accessing oral health care facilities are some of the factors responsible for the overall anosognosic disposition of patients. As a result, there is a tendency for overtreatment as an attempt to improve cure rates which ultimately increases associated post treatment morbidity of patients and undermines their quality of life (4,5).

Over treatment may also be a result of improper staging of oral cancer patients. Clinical methods used for staging are not sufficient to form the basis for treatment planning and adjunctive imaging modalities must be considered wherever possible to avoid under or over estimation of dimensions. Reliable indicators of locoregional recurrence would also greatly improve survival rates in OSCC patients (6). Despite the progress made in early detection and therapy, early predictors of cancer recurrence at the time of diagnosis are still missing for oral cancer.

Tumour associated tissue eosinophilia (TATE) has been reported in diverse sites (7-14) including the head and neck region and has been used as a prognosticator for many human malignancies (15-28). While its presence has been associated with a good prognosis in some studies, (15,23,25) others claim it is an indicator of poor prognosis in head and neck cancer (16,20,21).

The aim of our study was to analyse TATE quantitatively along the invasive front of the tumour in histopathological specimens of OSCC patients and correlate it with clinical staging, recurrence patterns and overall prognosis. It has already been established that ultrasonography (USG) gives an accurate measure of tumour dimensions comparable with histological measurements (29). To avoid the significant post-operative morbidity caused by stage migration, we first confirmed cTNM staging of the tumours with ultrasonographic findings before TATE analysis.

## Material and Methods

-Patients:

14 patients undergoing surgical resection for primary OSCC at M.S. Ramaiah Medical Hospital between January 2009 to January 2010 were selected for the study out of a total 45 patients initially screened, upon fulfilment of inclusion and exclusion criteria. A detailed case history was recorded, clinical examination and necessary investigations were performed and cTNM staging for the tumours was documented.

Patients with primary OSCC confirmed by biopsy who had not undergone radiotherapy, chemotherapy, or other any treatment prior to surgery were included in the study. Meanwhile patients with primary tumours at other anatomic locations, unresectable tumours and difficulty in mouth opening were excluded from the study. A major requirement was the availability of tumour tissue for microscopic analysis obtained from the invasive tumour front (ITF). Thus, incisional biopsies were excluded from the study. Clinical follow-up was maintained including data on locoregional recurrence and death. Duration of follow up was from January 2009 to December 2013.

-Ultrasonographic examination:

A grey scale USG evaluation (GE Volusun 350 at 7.5MHz) of the primary tumour was performed with an intra-oral probe to assess its transverse dimension and identify the appropriate biopsy site.

An incisional biopsy was performed to confirm the diagnosis following which the patients underwent surgical treatment.

**Figure 1:**
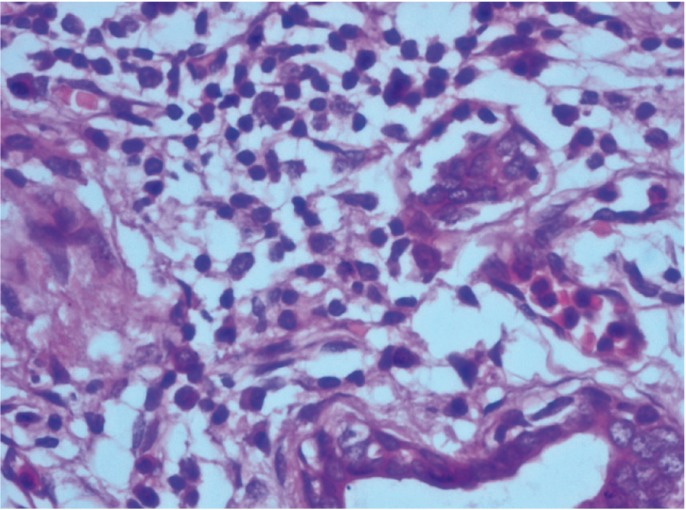
Mild eosinophilia (under 400x magnification).

**Figure 2:**
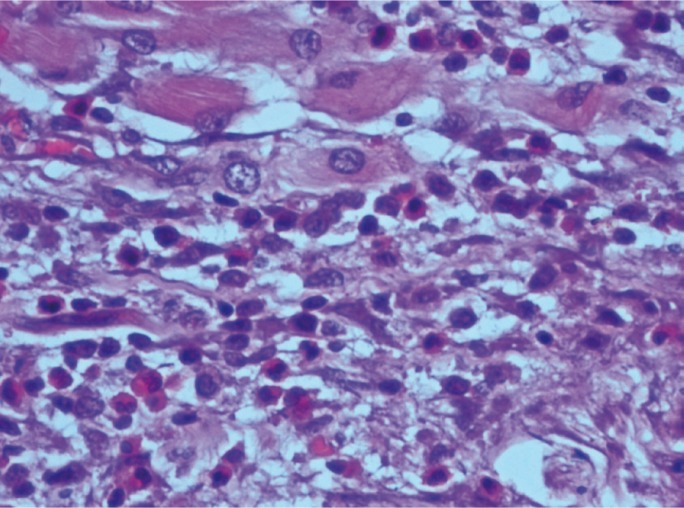
Moderate eosinophilia (under 400x magnification).

**Figure 3:**
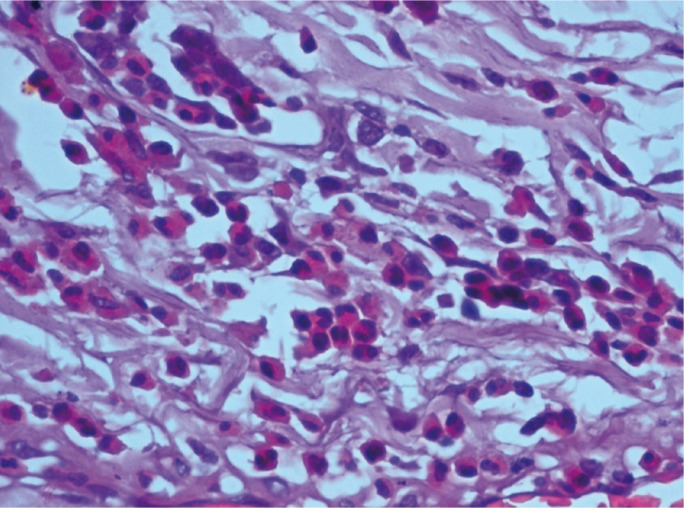
Intense eosinophilia (under 400x magnification).

-TATE analysis:

Paraffin embedded blocks of the tumour tissue were obtained from the archives of the Department of Pathology, M.S. Ramaiah Medical Hospital, Bangalore, India and the block representing the invasive front of the tumour was identified for each patient. 3µ sections were obtained and stained routinely with Haematoxylin and Eosin stain for TATE analysis. Eosinophils at the invasive front were counted under a high power objective (x 400) for 10 continuous high power fields (HPF) using a light microscope (OLYMPUS C-20 i). The mean values obtained were then graded according to the criteria given by Goldsmith et al.25 In this criteria, score ‘0’ corresponded with absence of eosinophils, ‘1+’ with presence of 5 to 10 eosinophils/HPF, ‘2+’with 10 to 20 eosinophils/HPF, ‘3+’ with 20 to 30 eosinophils/HPF and ‘4+’ with more than 30 eosinophils/HPF. We considered scores ‘0’ and ‘1+’ as absent/mild eosinophilia, scores ‘2+’ and ‘3+’ as moderate eosinophilia and ‘4+’ as intense eosinophilia, (Figs. 1-3).

## Results

The association between clinical staging, USG staging, locoregional recurrence and TATE has been summarized in  Table 1.

**Table 1:**
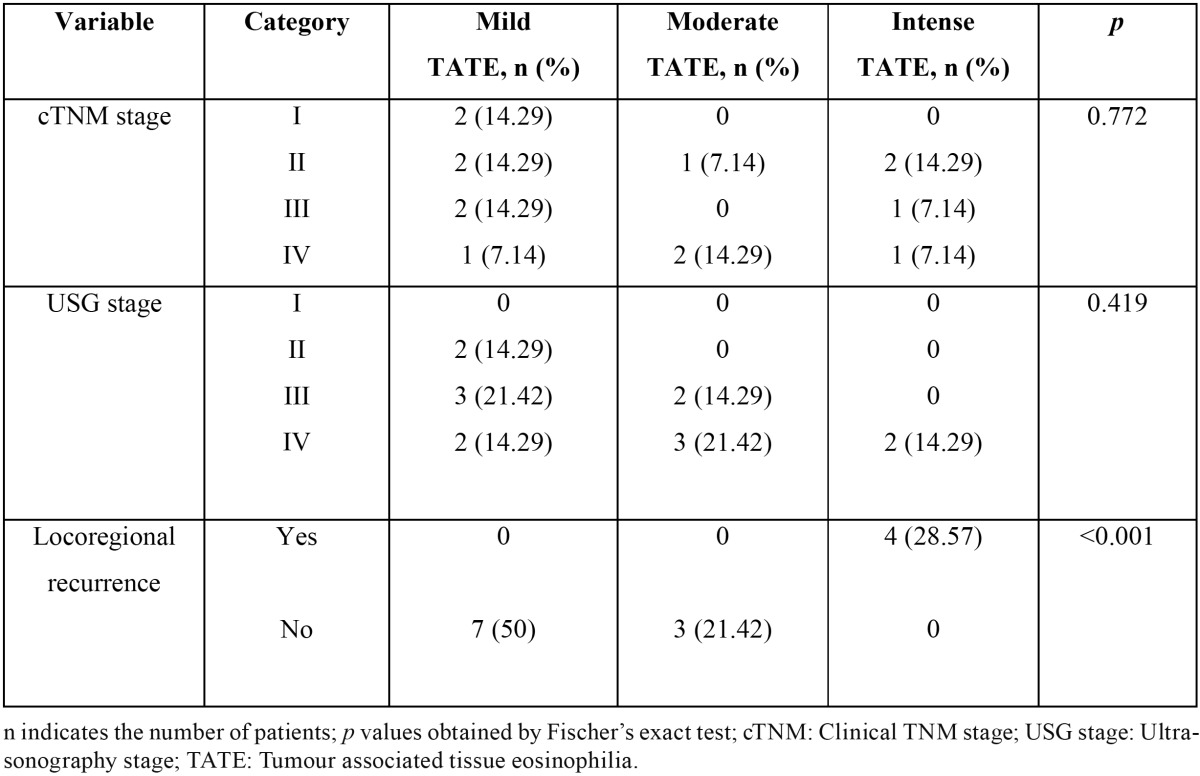
Diagnostic clinical manifestations of cGVHD (10).

**Figure 4:**
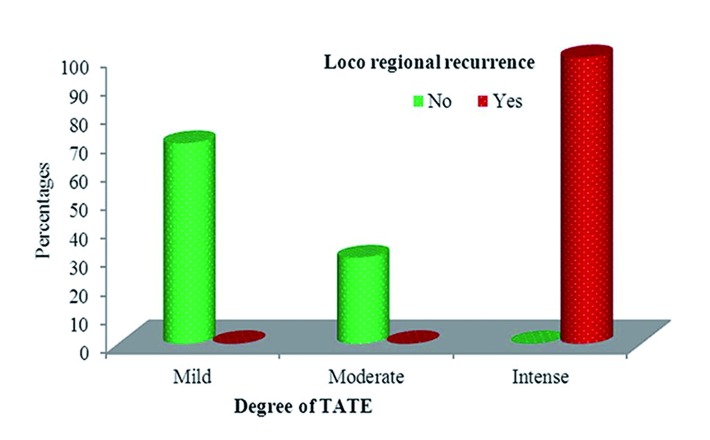
Association between degree of TATE and locoregional recurrence. All patients who developed intense TATE belonged to the ‘intense TATE’ category (*p*<0.001).

64.3% of the patients were female and 35.7% were male. 92.9% of the patients were aged more than 50 years with an overall age range of 45-76 years. Although, the clinical stage of these patients was underestimated in 10 cases (71.4%) when compared with USG findings, there were no statistically significant differences between the cTNM and USG stages with *p*=0.131. 7 cases (50%) demonstrated mild TATE, 3 cases (21.4%) demonstrated moderate TATE and the remaining 4 (28.6%) cases showed intense TATE. None of the patients with mild/moderate TATE showed locoregional recurrence. Loco regional recurrence was significantly associated with intense degree of TATE with *p*<0.001 ( Table 1). These patients were evaluated for TATE after surgical removal of the recurrent lesion. All of the 4 specimens showed higher values of TATE than the corresponding primary OSCC specimens with no change in overall degree of TATE. cTNM stage as well as USG stage did not correlate with the degree of TATE with *p*=0.419 and 0.772 respectively (Fig. 4,  Table 2, Table 3).

**Table 2:**
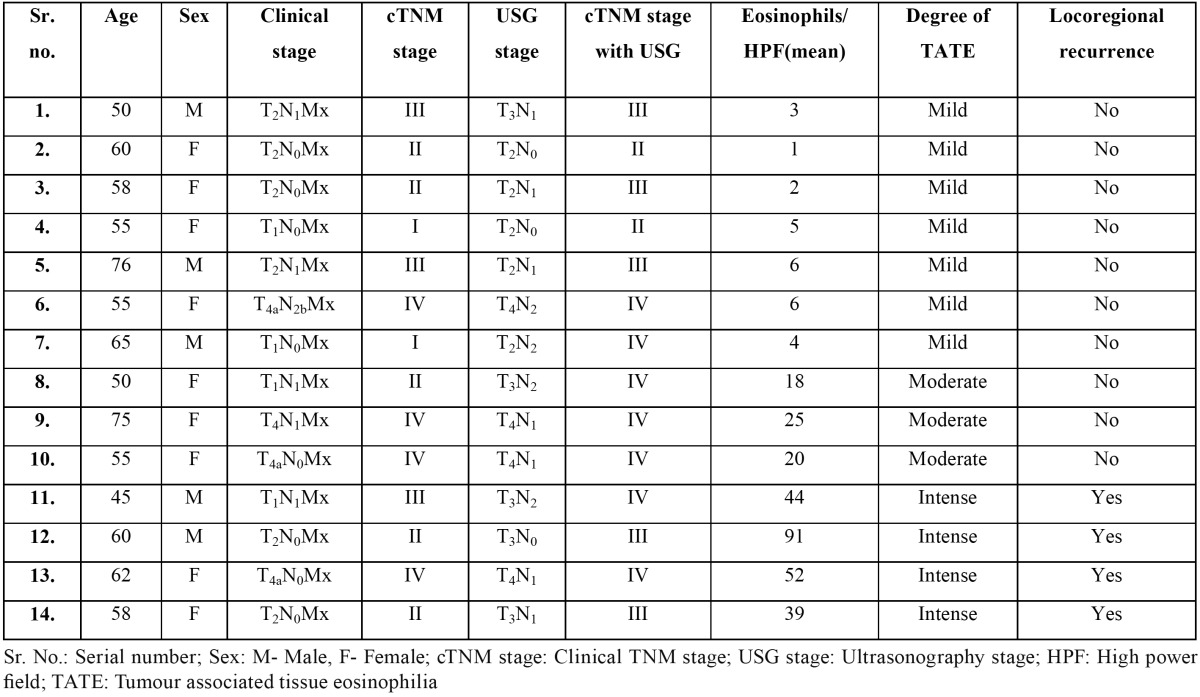
Summary of clinical data and study parameters.

**Table 3:**
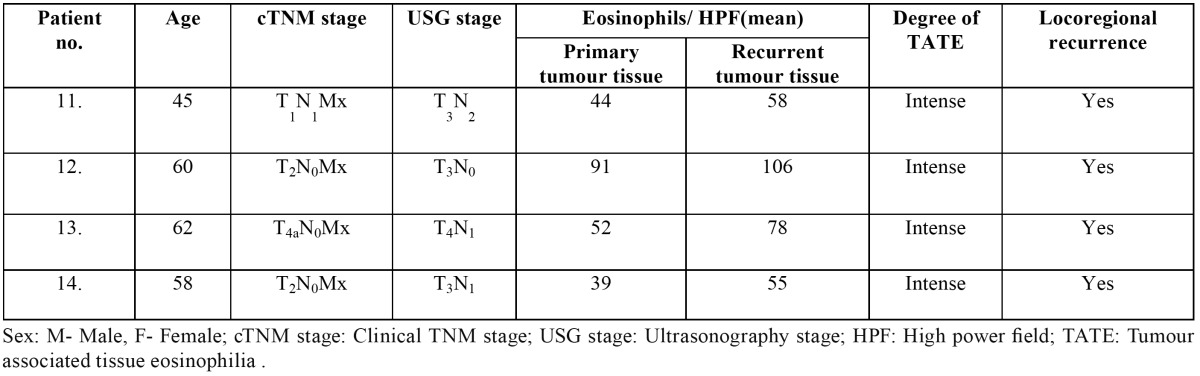
TATE values in patients who developed locoregional recurrence.

## Discussion

The complexities of the interactions between carcinomatous tissue and the underlying stroma has been an area of prime focus in cancer research with various features such as increased angiogenesis, presence of varying degrees of inflammatory infiltrates, desmoplastic stromal reaction and microinvasion recognized as important indicators of the general biology and behaviour of the tumour cell population (20).

The invasive tumour front (ITF) provides a ‘fertile ground’ for the assessment of such interactions as it houses the most aggressive cells of the tumour population, readily demonstrates molecular events of importance in tumour spread and is also less differentiated than the residual parts of the tumour (29). The ITF has gained a lot of attention in the past decade as an important prognosticator of human malignancies.

Eosinophils are bone marrow-derived, tissue-dwelling granulocytes found transiently in the blood circulation en-route to tissue inflammatory sites. They are mainly associated with parasitic infections and allergic diseases but are also known to participate in tissue remodelling and modulation of the host immune response (30). Their presence in intra and peri-tumoural sites has been well documented in a number of locations such as larynx, pharynx, esophagus, skin, breast, lung, intestine, genitourinary tract and also the oral cavity (16). Although mononuclear cells, and to a lesser extent neutrophils, are also found in oral cancers, eosinophils when present, form the predominant inflammatory cell population (30). However their role as well as association with prognosis of these tumours remains controversial.

Many have theorized eosinophils to have a tumour protective role due to secretion of cytotoxic mediators such as major basic protein, eosinophil cationic protein and eosinophil peroxidase and their ability to increase permeability of tumour killing cytokines into tumour cells (31). On the other hand, those who believe TATE to promote tumour growth have attributed this property to the active release of 92- kd gelatinase (of the matrix metalloproteinases family) which is involved in the breakdown of the basement membrane and the extracellular matrix (20). Also, a recent observation states that PGE2 which is expressed by OSCC and associated with its invasive nature shares its precursor with a potent eosinophil chemotactic molecule, PGD2 (31).

In this study we attempted to analyze TATE along the ITF of patients with OSCC. The patients were preoperatively subjected to a grey scale USG examination to determine the tumour dimensions and were compared with clinical measurements. A number of studies have validated the accuracy of tumour size assessment using USG (29). The transverse dimensions of the tumour obtained using USG were found to be greater than that measured clinically in 50% of the cases, as a consequence of which the cTNM stage had to be changed. We observed a strong association between intense TATE and locoregional recurrence. Interestingly, for the patients that did develop second primaries, the lesion was surgically excised and also analysed for TATE. All of these specimens showed a definite increase in the mean TATE/ HPF than that observed in the corresponding primary OSCC tissues. The patients are still being followed up on a regular basis.

Previous reports have found significant association between the presence of high degrees of TATE and stromal invasion in OSCC (20,31). An increased possibility of locoregional recurrence was also reported in such patients by Alrawi *et al.* (20). A recent study has even established the reliability of TATE as a reliable predictor of occult lymph node metastasis in clinically N0 OSCC patients (28). On the contrary, Dorta *et al.* (16) and Lorena *et al.* (32) did not find such an association.

A plausible explanation for this disparity in results of various studies could be due to the lack of a standard criteria for grading TATE that is universally followed, (16) use of biopsy specimens that run the risk of being unrepresentative, inadequacy of clinical methods alone in determining the tumour stage and the general lack of long term follow up of patients. In our study we included only those specimens that were obtained after surgical resection, where the invasive front of the tumour could be identified. We also confirmed clinical staging with ultrasonography and observed a 5 year follow up period for each of the patients.

Although intact eosinophils are identified with relative ease in routine H & E stained sections of tissue, it may get difficult to identify these cells in inflamed or fibrous tissue which is where special techniques such as autofluorescence and immunohisto-chemistry are recommended. Lorena *et al.* however reported the H & E technique to be as reliable as the immunostaining technique for identification of eosinophils. 32 In our study, eosinophils were easy to identify on H & E sections of 3 µ thickness but were often obscured in 4-5 µ sections due to overlapping of structures. Thus we studied only 3 µ sections.

Locoregional recurrence develops much earlier than metachronous disease and carries the worst prognosis. Patients who survive a first encounter with this disease have up to a 20 fold increased risk of developing a second cancer. In developing countries like India, OSCC is more prevalent among low socioeconomic status groups. Such patients are often lost to follow up and do not comply with lifestyle modifications. Thus, early identification of recurrence at the time of diagnosis will be useful in identifying patients at risk and employing customized treatment strategies and follow up policies for them (11,12).

The use of adjunctive imaging modalities such as USG to confirm the most appropriate biopsy site that should include the advancing front of the tumour or ITF may be helpful as TATE analysis can be done even in biopsied tumour tissue in such cases and based on the results, individualized treatment plans can be formulated. This method is simple, cost effective and can be performed routinely. Further studies recruiting larger samples are required to validate the findings.
